# Development of synthetic high-density lipoprotein-based ApoA-I mimetic peptide-loaded docetaxel as a drug delivery nanocarrier for breast cancer chemotherapy

**DOI:** 10.1080/10717544.2019.1618420

**Published:** 2019-07-10

**Authors:** Miaomiao Gong, Qi Zhang, Qi Zhao, Jiani Zheng, Yue Li, Siling Wang, Yue Yuan

**Affiliations:** a School of Pharmacy, Shenyang Pharmaceutical University, Shenyang, P. R. China;; b Shenyang Key Laboratory of Functional Drug Carrier Materials, Shenyang Pharmaceutical University, Shenyang, P. R. China;; c Department of General Surgery, General Hospital of Benxi Iron and Steel Co. Ltd, Benxi, P. R. China

**Keywords:** Synthetic high-density lipoprotein, mimetic peptide, docetaxel, breast cancer, anticancer

## Abstract

In this study, a synthetic high-density lipoprotein (sHDL), peptide-based nanocarrier loaded with docetaxel (DTX) was constructed, against breast cancer. The thermodynamic and molecular dynamic analyses were conducted to examine the stability of nanoparticles synthesized from mimetic peptide 5 A and various types of phospholipids. Furthermore, the cellular uptake and *in vivo* fluorescence imaging analysis experiments, with scavenger receptor B-I (SR-BI) were carried out to examine the tumor-targeting ability of sHDL. The nanoparticles were investigated for their pharmacodynamic and cytotoxic effects to show their effectivity as anti-tumor agents. The results showed that the synthesized sHDL nanoparticles exhibited a high payload of DTX, sustained drug release properties, and excellent biocompatibility. Moreover, DTX-sHDL nanoparticles enhanced the uptake of DTX, increased the cytotoxicity against MCF-7 cells, and reduced the off-target side-effects to normal cells. Finally, experiments in 4T1 cell line-bearing mice indicate that inhibition of tumor growth by DTX-sHDL nanoparticles was superior to that of free DTX group. Thus, the sHDL nanoparticles are a promising drug delivery vehicle for improving the efficacy of anti-cancer drugs.

## 1. Introduction

Breast cancer (BC) is currently a prevalent cancer, threatening physical and mental health of women all over the world (Dong et al., 2018). Applying chemotherapeutic drugs, such as taxanes is the preferred choice of treatment for women with early-stage or locally advanced breast cancer (Cánovas et al., 2018; Li et al., 2018). However, the chemotherapeutic benefits of taxanes are limited due to their poor solubility(Cho & Jung, 2018), serious toxicity (Gu et al., 2013), and non-tissue specificity (Joshi et al., 2014). Thus, taxanes cause harsh side effects due to the off-target effects on peripheral (normal) tissues (Zhang et al., 2018). Advances using nanotechnology for targeted drug delivery systems have shown great potential to overcome these challenges, thus leading to the development of effective chemotherapy (Deng & Zhang, 2018; Reza et al., 2018).

Currently, taxane-based drugs are developed in different nano-preparations, such as liposomes (Lv et al., 2018), copolymer micelles (Yao et al., 2017), and inorganic nanoparticles (Zhang et al., 2016) to reduce the side effects. Paclitaxel liposomes are employed clinically, but, their short half-life limits its wider application. The circulation time, *in vivo,* for normal liposomes is less than 30 min (Masayuki, 2014; Shah et al., 2017). Although PEGylation can extend the circulation time of Paclitaxel liposomes, it may lead to accelerated blood clearance (ABC) effects and safety concerns (Wang et al., 2015). The inorganic nanoparticles, without any modification, lack specific receptors *in vivo* (Cai et al., 2017), cannot be degraded and eliminated by the reticular endothelial system (Feliu et al., 2016). Furthermore, application of copolymer micelles is also limited for lack of intrinsic targeting property (Lee et al., 2010). Compared with the above-said synthetic nano-vehicles, synthetic high-density lipoprotein (sHDL) shows some unique properties that make it more effective to deliver drugs to specific targets.

sHDL, consisting of lipoprotein or mimetic apoA-I peptide (Uehara et al., 2015) and phospholipid molecules, exhibits many traits analogous to endogenous HDL (Rink et al., 2018). The extraction of endogenous HDL from human plasma is costly and laborious (Henderson et al., 2016). Thus, sHDL, composed of easily manufactured mimic peptides, holds significant potential for mediation of drug delivery (Rui et al., 2017). sHDL is a bionic and safe nanocarrier, which is completely biocompatible and biodegradable *in vivo*. sHDL has a long half-life and is stable even after long-term exposure to serum (Wang et al., 2018). sHDL nanoparticles typically range from 8 to 30 nm, in diameter (Jie et al., 2017); the ultra-small size, with large surface area, potentially enables sHDL to better transport the cargo via penetration or diffusion into target organs/tissues (Kuai et al., 2016). sHDL can target the SR-BI receptor, deliver cargo directly into the cytoplasm, and escape the endosome/lysosome pathway (Johnson et al., 2017). It has been reported that scavenger receptor B-I (SR-BI) receptor can specifically bind apoA-I on sHDL (Song et al., 2015). The 5 A peptide, synthesized by replacing the 37 pA mimetic peptide with 5 alanine residues (Stoekenbroek et al., 2015), resembles the amphipathic helices of apoA-I and can be used as a less cytotoxic therapeutic agent (Amar et al., 2010). Hydrophobic drugs can be inserted into the core of sHDL nanoparticles and delivered to the cellular (Yuan et al., 2016). According to previous reports (Shuo et al., 2013; David et al., 2015; Chang et al., 2017; Li et al., 2017), SR-BI is overexpressed in many types of cancer cells (breast, lymphoma, prostate, and ovarian), making its recognition a good platform for HDL-mediated targeted cancer drugs.

**Scheme 1.**
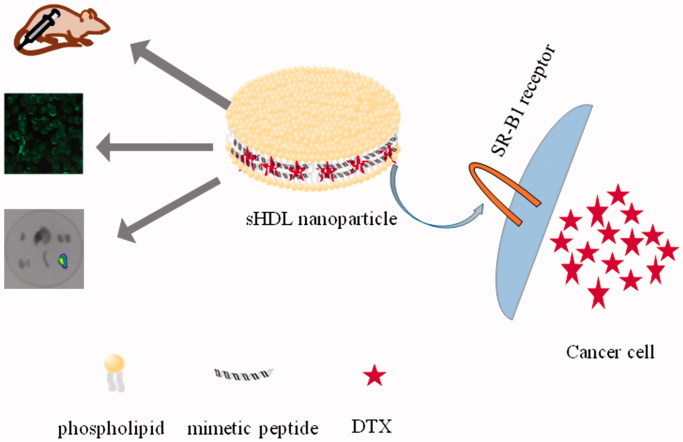
sHDL, composed of phospholipids and mimetic peptide, can specifically target to SR-B1 receptor and deliver hydrophobic cargo directly into the cytoplasm of cancer cells.

This study aimed to evaluate the effectiveness of sHDL-mediated drug delivery that utilizes the SR-BI gateway for treatment of breast cancer (shown in Scheme 1). In this study, phospholipids and mimetic peptide (5 A) could self-assemble into nanoparticles in an aqueous solution, via the process of thermal-cold cycling; thermodynamic and molecule dynamic analyses were conducted to screen the optimum phospholipid. Afterwards, hydrophobic chemotherapeutic, DTX, was loaded into the lipid layer of sHDL nanoparticles and the physicochemical properties of DTX-sHDL were investigated. To evaluate the specific targeting behavior MCF-7 breast cells, overexpressing SR-BI, and HaCaT human normal epithelial cells were used as *in vitro* models for cellular uptake experiments. Additionally, fluorescence imaging was employed to examine the *in vivo* targeting ability of sHDL nanoparticles, in tumor-bearing mice, compared to normal mice. Cytotoxicity and pharmacodynamics experiments were also performed to determine the effectiveness of the sHDL nanoparticles against tumor *in vitro* and *in vivo*. This biomimetic delivery system possesses an active targeting mechanism, through which DTX was delivered specifically into cancer cells, to achieve anti-cancer response.

## 2. Materials and methods

### 2.1. Materials

Docetaxel (>99% purity, DTX) was obtained from Dalian Meilun Biotechnology Co. Ltd. 1, 2-dipalmitoyl-sn-glycero-3-phosphocholine (DPPC), 1-palmitoyl-2-stearoyl-sn -glycero-3-phosphocholine (HSPC), 1-palmitoyl-2-oleoyl-sn-glycero-3-phosphocholine (POPC) and 1, 2-dimyristoyl-sn-glycero-3-phosphocholine (DMPC) were donated by A.V.T (Shanghai) pharmaceutical Co. Ltd. Synthesized 5 A was purchased from GenScript. Fluorescein isothiocyanate (FITC), 3-(4, 5-dimethyl-thiazol-2-yl)-2, 5-diphenyl tetrazolium bromide (MTT) was obtained from Sigma. All other chemical reagents of analytical purity were acquired from commercially available sources.

### 2.2. Preparation of sHDL nanoparticles

Thin-film dispersion method was used to prepare blank sHDL nanoparticles. Briefly, 15 mg of phospholipids (DMPC, DPPC, HSPC, and POPC) were dissolved in 2 mL chloroform, and the organic solvent was removed by rotary evaporation, followed by vacuum drying to remove any residue. Afterwards, 0.7 mL PBS (pH 7.4) was added to hydrate the film and 0.3 mL was added to dissolve 5 mg of 5 A peptide using probe-type ultra-sonicator. The mixture was heated in the water bath for 10 min, cooled below phase transition temperature for 10 min, and circulated for 3 times to form DMPC-sHDL, DPPC-sHDL, HSPC-sHDL, and POPC-sHDL nanoparticles.

### 2.3. Physicochemical characterization

#### 2.3.1. Particle size, turbidity and appearance

Particle sizes of DMPC-sHDL, DPPC-sHDL, HSPC-sHDL and POPC-sHDL nanoparticles were gauged by Dynamic Light Scattering (DLS) analyzer (Zetasizer 3000 HAS, Malvern, UK) at 23 °C with a 90° scattering angle. The turbidity of all samples was measured using UV-Vis light at a wavelength of 480 nm.

#### 2.3.2. Differential scanning calorimetry (DSC)

The experiments were carried out with 20 μL of prepared sHDL nanoparticles in hermetically sealed aluminum pans (Hugo et al., 2018). The DSC cell with the samples was equilibrated at −40 °C, and the temperature was increased to 30 °C at a rate of 5 °C per minute with a sealed blank pan for comparison. The peak temperature (*T*p) was recorded, and reaction heat was calculated by the peak area.

#### 2.3.3. Molecular dynamics

The structure of 5 A (Xianglan et al., 2011) (DWLKAFYDKVAEKLKE AFPDWAKAAYDKAAEKAKEAA) was obtained from SWISS-MODEL, and the structures for DMPC, DPPC, HSPC, and POPC were generated by Chemdraw. The initial energy of solvated complex was minimum under solute constraint as well as the unconstrained complete energy. Each energy minimization involved a 5000 step steepest descent, and then another 1,000 step conjugate gradient (Wen et al., 2016). Subsequently, 100 ps MD simulation with position constraint was performed for the complex. The integrated system was heated from 0 to 300 K by using 100-ps molecular dynamics simulation. Finally, berendsen weakly-coupled algorithm was used to simulate the 10 ns unconstrained NPT with temperature and pressure controlled at 300 K and 1 atm respectively, to gain the binding energy (Zhang et al., 2017).

### 2.4. Preparation and characterization of DTX-sHDL

#### 2.4.1. Particle size, morphology, EE and DL

15 mg of DMPC were dissolved in 2 mL chloroform, 0.6 mg DTX was added to the DMPC, and the organic solvent was removed by rotary evaporation, followed by vacuum drying to remove any residue. Afterwards, 0.7 mL PBS (pH 7.4) was added to hydrate the film and 0.3 mL was added to dissolve 5 mg of 5 A peptide using probe-type ultra-sonicator. The mixture was heated in the water bath for 10 min, cooled below phase transition temperature for 10 min, and circulated for 3 times to form the DTX- sHDL nanoparticles.

Dynamic Light Scattering (DLS) Analyzer (Zetasizer 3000 HAS, Malvern, UK) was used to evaluate particle size of DTX-sHDL.

DTX-sHDL was diluted appropriately, placed on a carbon-coated copper wire, stained with 2% (w/v) phosphotungstate solution, and dried at room temperature. The morphology of DTX-sHDL was visualized by JEOL-100 CX II transmission electron microscopy (TEM).

Any un-encapsulated DTX was separated from preparations using a microcolumn. 0.2 mL solution of DTX-loaded HDL nanoparticles were added to a 2 mL microcolumn and centrifuged at 2000 rpm to collect the eluant. The concentration of DTX was measured by high performance liquid chromatograph (HPLC). The chromatographic conditions were Diamonsil C18 column (4.6 mm × 150 mm, 5 μm), acetonitrile: water (55:45, v/v) at 1.0 mL/min, 230 nm detection wavelength. The DTX encapsulation efficiency (EE) and drug loading (DL) of DTX-sHDL nanoparticles was calculated as per the following formula:

$$\text{EE}\%={\text{m}}_{\left(\text{DTX in nanoparticles}\right)}/{\text{m}}_{\left(\text{Original DTX}\right)}*100\%$$

$$\text{DL}\%={\text{m}}_{\left(\text{DTX in nanoparticles}\right)}/{\text{m}}_{\left(\text{nanoparticles}\right)}*100\%$$

#### 2.4.2. Release behavior of DTX from sHDL nanoparticles

The release study of DTX *in vitro* was conducted by dialysis, the release medium was PBS (pH 7.4) containing 0.1% Tween 80. The nanoparticles were tightly sealed and placed in a dialysis bags (MWCO: 1 kDa), and soaked in release medium, at 37 °C, under constant stirring. The release medium (1 mL) was removed and replenished with equal volume of fresh medium, at certain time intervals. The samples were monitored by HPLC to obtain the release extent of DTX.

### 2.5. *In vitro* cytotoxicity assay

The cytotoxic effect of sHDL nanoparticles was evaluated by the 3-(4, 5-dimethyl- thiazol-2-yl)-2, 5-diphenyl tetrazolium bromide (MTT) assay with MCF-7 cells (SR-BI positive) and HaCaT cells (SR-BI negative). The cells were seeded in a 96-well plate at a density of 1.0 × 10^4^ cells per well and incubated for 12 h at 37 °C. After discarding the medium, the cells were treated with 200 μL of free DTX, blank sHDL and DTX-sHDL at several concentrations for 48 h at 37 °C with 5% CO_2_. Afterwards, the cells in each well were incubated with 20 μL MTT solution (5 mg/mL) for 4 h. Thereafter, the medium containing MTT was removed and then 200 μL dimethyl sulfoxide (DMSO) was added to dissolve formazan. The content of formazan was determined by the UV at 560 nm. Cytotoxicity was expressed by the absorbance ratio between treated and untreated cells.

### 2.6. *In vitro* intracellular uptake

To distinctly visualize the endo-cellular behavior of sHDL nanoparticles and the targeting property of SR-BI, the peptide was replaced with a FITC-labeled peptide for fluorescence imaging. The cellular uptake of sHDL nanoparticles was analyzed by confocal laser scan microscopy (CLSM) and flow cytometer (FCM). The MCF-7 cells and HaCaT cells were seeded in 24-well plates. After incubation for 12 h, the culture medium was removed and replaced by FITC marked blank sHDL and DTX-sHDL. After 4 h, the samples were washed with cold PBS 3 times and fixed with paraformaldehyde for 15 min. The CLSM was used to observe the fluorescence signals within fixed cells. In addition, after 4 h of attachment, the cells were resuspended in PBS and measured with FCM.

### 2.7. *In vivo* anti-tumor efficacy

All animal experiments were conducted in strict accordance with the guidelines for Animal Experimentation of Shenyang Pharmaceutical University (Shenyang, China) and the protocol was approved by the Animal Ethics Committee of the Institute (no. SYXK (Liao) 2014-0004). The *in vivo* anti-cancer efficacy of the preparation was evaluated in KM mice inoculated with 4T1 cells. When the tumor reached ∼100 mm^3^, KM mice (25 ± 2 g, female) were randomly divided into four groups (*n* = 6) and intravenously administered (through the tail vein) saline, free DTX, blank sHDL or DTX-sHDL nanoparticles with equivalent doses of DTX, once every two days, for 10 days. The tumor sizes and body weights were measured every other day before dosing. At day 12, all the mice were sacrificed; their tumors as well as other organs (liver and heart) were resected, weighed, and fixed with formalin. The fixed organs were embedded in paraffin blocks and stained with hematoxylin and eosin (H&E), to be observed by optical microscope. The tumor growth inhibition rate was calculated as per the following formula:

Inhibition(%)=(C−T)/C×100

where *C* and *T* were the average tumor weights of the control group and the treated groups, respectively.

### 2.8. *In vivo* fluorescence imaging

For *in vivo* fluorescence imaging, DTX-sHDL nanoparticles were labeled with NIR dye DiR and injected into the tail vein of KM mice, inoculated with 4T1-tumor, to research the biodistribution and tumor-targeting ability. The *in vivo* FX Pro imaging system was applied to process the fluorescence imaging data, collected at predetermined time intervals: 2 h, 6 h, 12 h, 24 h, and 48 h. The images were analyzed using a Carestream Molecular Imaging software (Kodak, Rochester, NY, USA). The wavelength of excitation and emission were 720 nm and 790 nm, respectively. After 48 h, the mice were sacrificed and tumor tissues and main organs were dissected for imaging.

### 2.9. Statistics analysis

All experiments were expressed as mean ± SD from experiments, performed at least in triplicate. Student’s *t*-test was used to statistically analysis the data, and *p* < .01 was considered statistically significant.

## 3. Results and discussion

### 3.1. Selection of the phospholipid composition of sHDL

Different types of phospholipids (Figure S1) affect the formation and stability of sHDL. Thus, it is necessary to select the optimal type of phospholipid to achieve the optimal properties. As shown in Table S1, the particle size of the blank DMPC-sHDL nanoparticles was 12.58 ± 0.34 nm with the polydispersity index (PdI) of 0.128, which is similar to that of endogenous HDL (8–14 nm) and could exhibit similar properties. The appearance of sHDL made up of the mimetic peptide and different types of phospholipids was significantly distinct. DMPC showed the lowest turbidity, while POPC showed the highest.

The cloudiness of the solution could be attributed to an inadequate amount of apoA-I mimetic in the lipid solution and the different transition temperatures of lipids (Figure S2). When the temperature was higher than the phase transition temperature, the phospholipid molecules will increase the fluidity and permeability of the membrane. While when the temperature was lower than the phase transition temperature, the phospholipid molecules will shrink to remain stable. The appropriate phase transition temperature was favorable for the combination of phospholipids and peptide to form stable nanoparticles. The transition temperatures of DMPC (*T*_m_ = 23 °C) was close to room temperature, which could provide a better trapping of mimetic peptide into the lipid bilayers to decrease peptide leakage or dissociation from membrane.

**Figure 1.**
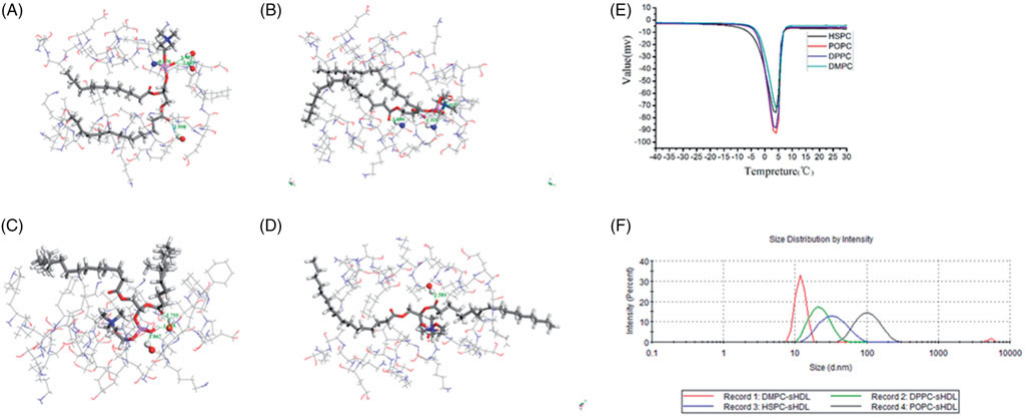
The molecular dynamic analysis models of mimetic peptid and (A) DMPC, (B) DPPC, (C) HSPC, and (D) POPC, respectively. (E) DSC curves of the four blank sHDL nanoparticles. (F) The DLS of sHDL nanoparticles.

To further prove the stability of different types of sHDL, we conducted molecular dynamic analysis that probe the affinity of the phospholipid to the mimetic peptide. In the models, all the key residues lined the channel pore, demonstrating that the two homologous models had satisfactory geometry. Intermolecular forces push the phospholipid and peptide to form the final structure. The hydrogen bond forms the driving force; the number of hydrogen bonds and distance, together, determine the force strength, namely the stability of sHDL (Figure 1(A–D)). The results showed that DMPC had the maximum value of the strength of force.

To prove the stability of molecules, we studied the thermodynamic process using DSC method, yielding the reaction heat value (Figure 1(E)). When examined under the same temperature and pressure of the system, the reaction heat value can indicate the stability of nanoparticles and indirectly indicate the binding ability of the phospholipids to the mimetic peptides. As the reaction is exothermic, the smaller the change in reaction heat, higher is the stability of the substrates. In summary, the stability result was: DMPC > DPPC > HSPC > POPC. Based on its size, clarity, turbidity, and stability, we chose 5 A and DMPC as the key components of sHDL for further investigation.

### 3.2. Characterization of DTX-sHDL

Altering the ratio of 5 A peptide and DMPC could potentially alter the diameter of the sHDL nanoparticles and its drug loading. To encapsulate the core of sHDL, a hydrophobic drug was utilized to fill it through physical binding. The optimization ratio of peptide-to-lipid also avoided the presence of both, lipid and free peptide, dispersions. The encapsulation efficacy and optimal drug loading of DTX-sHDL nanoparticles, 1:3 (w/w) peptide-DMPC with a 3% theoretical loading, analyzed by HPLC method was 66.5% and 2.01%, respectively, indicating that the hydrophobic DTX was efficiently loaded into sHDL nanoparticles.

**Figure 2.**
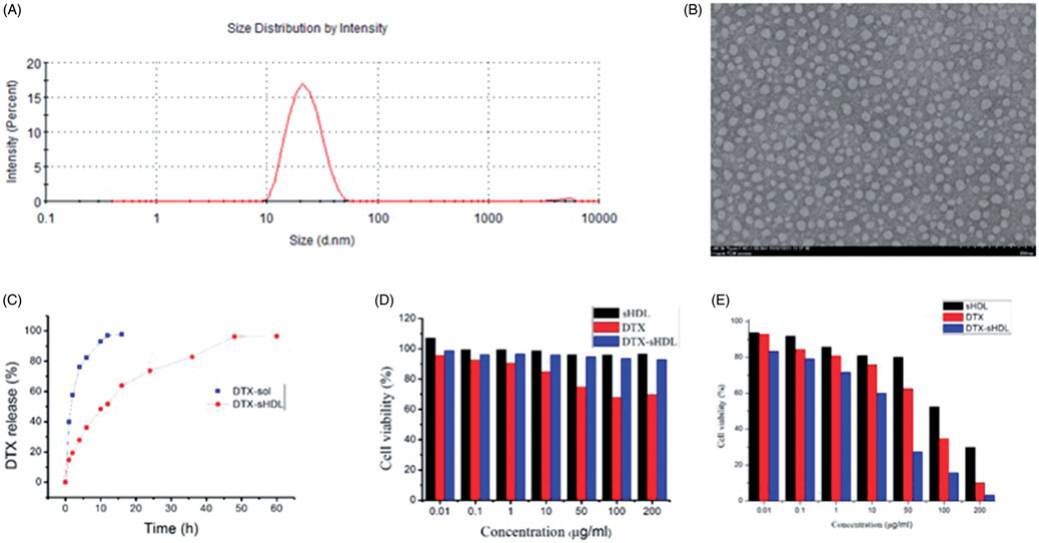
(A) DLS image of DTX-sHDL nanoparticles. (B) TEM image of DTX-sHDL nanoparticles. (C) DTX release profiles from DTX-sol and DTX-sHDL nanoparticle in pH 7.4 PBS. Cell viability of sHDL, DTX and DTX-sHDL nanoparticle against (D) HaCaT cells and (E) MCF-7 cells at different concentrations, respectively. Cytotoxicity of results were expressed as the mean ± SD from three independent experiments (*n* = 6).

The average diameter of 20 nm with a narrow size distribution of DTX-sHDL was determined by DLS, and the homogeneous display with discoidal shape of the optimal formulation, caused by favorable lipid binding properties of ApoA-I mimetic peptides, was examined by TEM (Figure 2).

The DTX-sHDL nanoparticles can be stored at 4 °C. After 10 days of storage, particle size and encapsulation efficiency hardly changed, while the encapsulation efficiency decreased significantly, from 66.5% to 52.6% after 20 days.

### 3.3. *In vitro* release of DTX in DTX-sHDL nanoparticles

To investigate the release behavior of DTX-sHDL nanoparticles *in vitro*, the nanoparticles and free DTX solution (DTX-Sol) were placed in isotonic PBS (pH = 7.4), containing 0.1% (w/v) Tween 80 (Figure 2(C)). Compared to DTX-Sol, DTX-sHDL nanoparticles exhibited a sustained release of DTX in pH 7.4 PBS. There was no burst release at 4 h, and the cumulative release was as high as 97% after 48 h, demonstrating that sHDL nanoparticles could generate the drug-sustained release behavior. The kinetic mechanism of releasing DTX from nanoparticles was studied by using the cumulative data of the release process fitted by zero-order and first-order equations, Higuchi model and riger-peppas model (Table S2). According to the fitting equation, the Riger–Peppas equation has the highest fitting degree, so it can be considered that the release mechanism of DTX in nanoparticles is the combination of Fick diffusion and skeleton disintegration.

### 3.4. *In vitro* cytotoxicity

To investigate the cellular cytotoxicity of the nanoparticles, HaCaT cells and MCF-7 cells were treated with blank sHDL, DTX-sHDL, and DTX-Sol. The viability of HaCaT cells treated with blank sHDL and DTX- sHDL nanoparticles, even at high concentrations was usually as high as 95% when compared to the cells treated with DTX-Sol (Figure 2(D–E)). This confirmed a good biocompatibility of the nanoparticles, for future use in drug delivery process. In addition, DTX-loaded nanoparticles showed appreciable cytotoxicity to MCF-7 cells and the cytotoxicity of DTX-sHDL nanoparticles was significantly higher than that of DTX-Sol. The half maximal inhibitory concentration (IC50) of DTX-sHDL nanoparticles (1.113 μg/mL) was about 26-fold lower than that of DTX-Sol (29.77 μg/mL), which demonstrated that the DTX-sHDL nanoparticles developed a foreseeable effect *in vitro* anti-tumor efficacy. Interestingly, blank sHDL nanoparticles also displayed certain anti-tumor activity against MCF-7 cells, which overexpressed SR-BI – the sHDL-specific receptor. The sHDL nanoparticles themselves were potent therapeutic agents, probably via cellular cholesterol depletion. This, the nanoparticles could reach a dual function against cancer cells, with a small dose having huge anti-cancer effect, and low cytotoxicity to normal cells. The physicochemical properties and targeted delivery of DTX make it an ideal transporter of anti-cancer agents.

### 3.5. *In vitro* intracellular uptake

To investigate the intracellular uptake ability of the sHDL nanoparticles, which possess a high affinity to malignant tumors overexpressing SR-BI receptors, CLSM and FCM were employed for qualitative and quantitative evaluation. We chose FITC-marked 5 A as a sensitive fluorescent marker, to confirm the direct fluorescent intensity distinction among different experiment groups.

**Figure 3.**
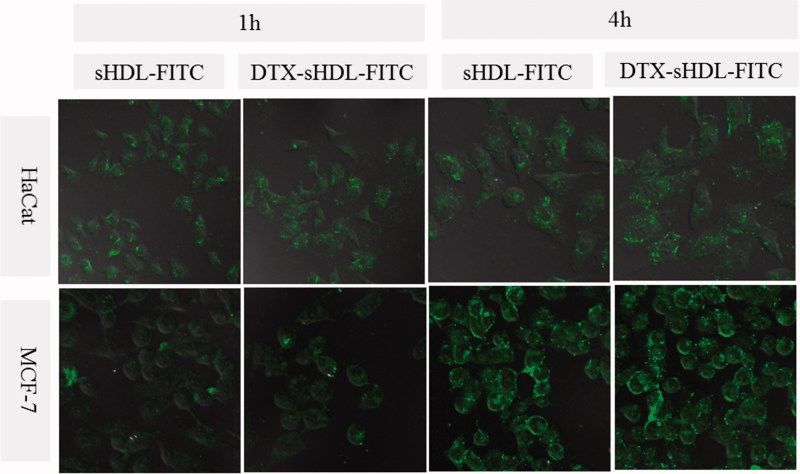
CLSM images of HaCaT cells and MCF-7 cells incubated with sHDL-FITC nanoparticle and DTXsHDL-FITC nanoparticle for 1 h and 4 h.

As shown in CLSM (Figure 3), all sHDL-FITC nanoparticles and DTX-sHDL-FITC nanoparticles exhibited a time-dependent FITC accumulation, in both HaCaT cells and MCF-7 cells. The fluorescence of sHDL-FITC nanoparticles and DTX-sHDL-FITC nanoparticles in MCF-7 cells was significantly stronger than that of their counterparts in HaCaT cells (for both 1 and 4 h incubations). The SR-BI receptor could specifically bind sHDL nanoparticles and the endothelial cells transcytosis the HDL required for the functions of SR-BI. Therefore, the MCF-7 tumor cells showed a high level of SR-BI expression and could uptake much more sHDL-FITC nanoparticles, than the HaCaT cells, which expressed much lower levels of SR-BI. These results indicated that sHDL nanoparticles are beneficial to promote the uptake in SR-BI overexpressed cells. When the nanoparticles encountered the cells, apoA-I in SR-BI bound sHDL, on the cell membrane, selectively brought the lipid components in lipophilic core of sHDL-based nanoparticles into the cytoplasm, through the SR-BI hydrophobic channel.

To quantitatively evaluate the cellular uptake of nanoparticles, intracellular FITC concentrations after different conductions were decided by FCM (Figure S3). The results of FCM were consistent with those through the CLSM observation. The intracellular FITC concentrations in MCF-7 cells were higher than those in HaCaT cells: the sHDL nanoparticles can exhibit a better anti-breast cancer effect. Meanwhile, although the low-expression cells can only uptake a few nanoparticles, the cytotoxicity is very little, which will be discussed in the next part.

### 3.6. *In vivo* anti-tumor efficacy

**Figure 4.**
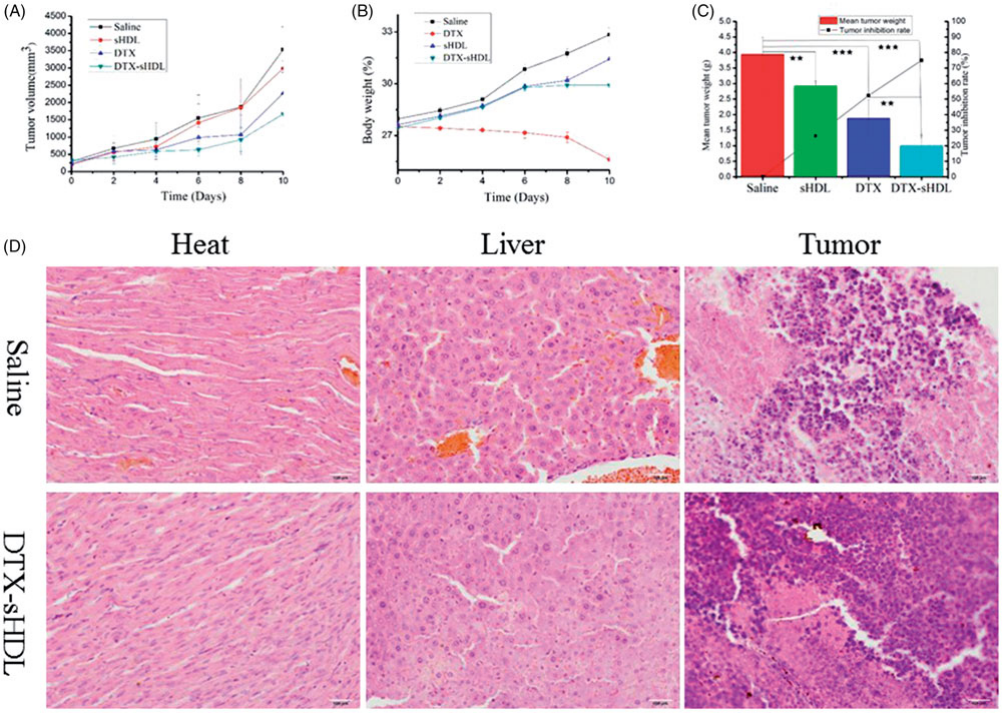
(A) Changes in body weight after tail intravenous injection of physiological saline, free DTX and DTX-sHDL nanoparticles (B) The tumor volume variations of every group. (C) The tumor weight and inhibition of mice after 10 days treatment. The tumor volume and mean tumor weight between groups was compared by Student’s t test *p < .1. (D) Hematoxylin and eosin (H & E) stained histological sections of major organs (heart, liver, and tumor) from mice treated with saline group as control and sHDL nanoparticle group.

To investigate the potential effect of DTX-sHDL nanoparticles for inhibiting tumor growth, an *in vivo* therapeutic efficacy analysis was conducted using KM mice with 4T1 breast cancer. The body weight and tumor size were constantly measured during a treatment period of 10 days. The body weight of the free DTX-treated group reduced noticeably, while other groups treated with physiological saline, sHDL and DTX-sHDL nanoparticles showed an opposite trend (Figure 4(A–C)), indicating that the incorporation of DTX into sHDL nanoparticles significantly reduces the toxicity of DTX. Besides, compared to the saline group, the average volume of tumors treated with free DTX and DTX-sHDL nanoparticles reduced, particularly DTX-sHDL showed a sharp reduction (Figure S5). This proved that the anti-cancer effect of nanoparticles was better than that of free DTX. Additionally, epidemiology suggested that low HDL level might cause cancer risk (Mathilde et al., 2015). Thus, the sHDL nanoparticles could overcome a weaker ability against tumors by increasing the number of HDL molecules *in vivo*. The tumor inhibition rates by the action of sHDL, free DTX and DTX-sHDL nanoparticles (26.03%, 52.31%, and 74.90%) were consistent with the tumor volume tendencies. In the cytotoxicity experiment, blank nanoparticles showed strong cytotoxicity to McF-7 cells at high concentrations. When the concentration reached 100 and 200 μg/mL, 50% and 30% of cells survived, respectively. The tumor inhibition rate of blank nanoparticles can reach 26.03% in the anti-tumor activity experiments, further proving the anti-tumor effect of blank carriers. We concluded that the encapsulated anti-cancer drugs with HDL express enhanced cytotoxic effects against cancer cells. The beneficial effects of DTX-sHDL nanoparticles were attributed to the ability of sHDL to deliver drug to specifically targeted tumor cells.

To investigate the latent toxicity of sHDL nanoparticles in mice, we analyzed the major organs (heart, liver, and tumor), histologically. Compared to the control group, no significant toxicity-related changes were observed after treatment with sHDL nanoparticles, via hematoxylin and eosin (H & E) staining sections (heart and liver) (Figure 4(D)). However, inflammation – a marker of tumor death, was observed in the tumors treated with nanoparticles, when compared to the control group. Thus, the sHDL nanoparticles express a strong anti-tumor effect. Collectively, these results suggest that sHDL nanoparticles have a good biocompatibility for *in vivo* treatment. Therefore, sHDL nanoparticles are a promising nano-vehicle for effective intracellular DTX delivery, good anti-tumorigenic abilities and lower side effects in cancer therapy.

### 3.7. *In vivo* fluorescence imaging

**Figure 5.**
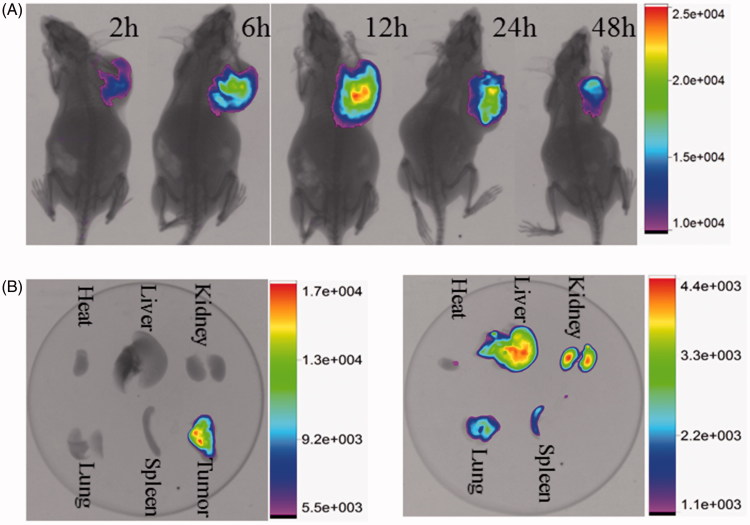
(A) The *in vivo* imaging of 4T1 tumor-bearing KM mice after administration of nanoparticles at 2 h, 6 h, 12 h, 24 h and 48 h. (B) The fluorescence images of tumor-bearing mice and normal mice isolated organs.

The biological distribution and accumulation of sHDL nanoparticles in tumor sites of KM mice, bearing breast cancer 4T1 cells, were carried out by *in vivo* fluorescence imaging. After the administration of sHDL nanoparticles, increasing amounts of fluorescent nanoparticles were collected at the tumor site, over time, continuously and efficiently, with the fluorescence intensity in the tumor site being observed even up to 48 h (Figure 5). This suggested that the nanoparticles have a favorable tumor targeting ability and long circulation time *in vivo*. We also found that the sHDL nanoparticle can reach the tumor location even 2-h postinjection, revealing a rapid uptake rate of the nanoparticle by the tumor. The fluorescence intensity of individual organs after dissection can illustrate the bio-distribution and organ accumulation of nanoparticles. As expected, no obvious fluorescence accumulation was in normal tissues, 12 h after administration. However, high intensity fluorescence, at the tumor site, could be observed during the circulation of the nanoparticles, as they bind the overexpressed SR-BI receptors rapidly and specifically. In contrast, the distribution of nanoparticles in normal KM mice (without tumor) was observed in the liver and kidney, because of the capture and elimination by RES. In general, the above results of *in vivo* biodistribution revealed that sHDL nanoparticle exhibits relatively long circulation half-life, strong tumor targeting ability after injection, and low toxicity of DTX due to the reduced off-target side effects to normal tissue. Thus, sHDL nanoparticles can be developed as an effective tumor-targeting drug delivery system.

## 4. Conclusions

In summary, the use of phospholipid and mimetic peptide 5 A as the vehicle to prepare sHDL nanoparticles has many significant merits: a simple synthesis route, biocompatibility, and active targeting via effective binding to SR-BI receptor. Thermodynamic and molecule kinetic analyses reveal that the sHDL nanoparticles formed by DMPC and 5 A can obtain an optimally stable formulation, increase the hydrophobic drug loading capacity, and achieve the sustained *in vitro* release in the physiological environment to realize relatively long circulation time. Compared to SR-BI-negative cells, the improved cellular uptake of DTX-sHDL nanoparticles in SR-BI-positive cells was verified by CLSM and FCM, confirming the higher targeting and anti-cancer abilities. Moreover, compared with free DTX, DTX-sHDL nanoparticles exhibit better inhibition of tumor growth and fewer side effects. The *in vivo* fluorescence imaging analysis explains the binding capability of tumor and sHDL nanoparticles and demonstrates further the anti-cancer ability of the nanoparticles. The specific delivery of drugs to tumor cells was facilitated via the SR-BI receptor, making the sHDL drug delivery system a distinct and feasible enhancement agent for cancer treatment. Therefore, particle function of sHDL nanoparticles is a main tool for HDL-based drug delivery strategies.

## Supplementary Material

supplyment.docx
