# Supplementary material for: Development of synthetic high-density lipoprotein-based ApoA-I mimetic peptide-loaded docetaxel as a drug delivery nanocarrier for breast cancer chemotherapy
Source: Drug Deliv. 2019 Jul 10;26(1):708–16. doi: 10.1080/10717544.2019.1618420 (PMC6691925; doi:10.1080/10717544.2019.1618420)
Supplement: supplyment.docx [file IDRD_A_1618420_SM3214.docx]

**Supplementary Information**

Development of synthetic high-density lipoprotein-based ApoA-I mimetic peptide loaded docetaxel as a drug delivery nanocarrier for breast cancer chemotherapy

Miaomiao Gong^1,2^, Qi Zhang^3^, Qi Zhao^1,2^, Jiani Zheng^1,2^, Yue Li^1,2^, Siling Wang^1,^*, Yue Yuan^1,2^*,

1School of Pharmacy, Shenyang Pharmaceutical University, 103 Wenhua Road, Shenyang, 110016, P. R. China

2Shenyang Key Laboratory of Functional Drug Carrier Materials, Shenyang Pharmaceutical University, 103 Wenhua Road, Shenyang, 110016, P. R. China

3Department of General Surgery, General Hospital of Benxi Iron and Steel CO. LTD, No. 29 Renmin Road, Benxi, 117000, P. R.China,

*Corresponding author: Prof. Yue Yuan and Prof. Siling Wang

Mail address: School of Pharmacy, Shenyang Key Laboratory of Functional Drug Carrier Materials, Shenyang Pharmaceutical University, No.103, Wenhua Road, Shenyang 110016, China

Tel: +86-24-43520585; Fax: +86-24-43520585

Mobile phone: 13940047880 (Yue Yuan)

E-mail: hiyueyuan@163.com (Yue Yuan), silingwang@syphu.edu.cn (Siling Wang)


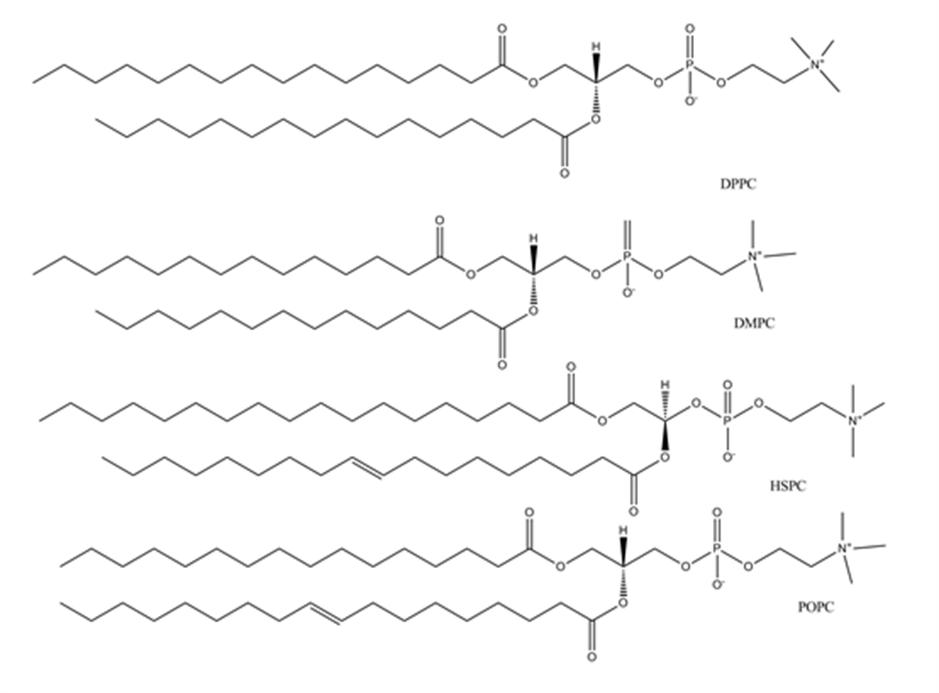


Figure S1. The structure of DMPC, DPPC, HSPC, and POPC.


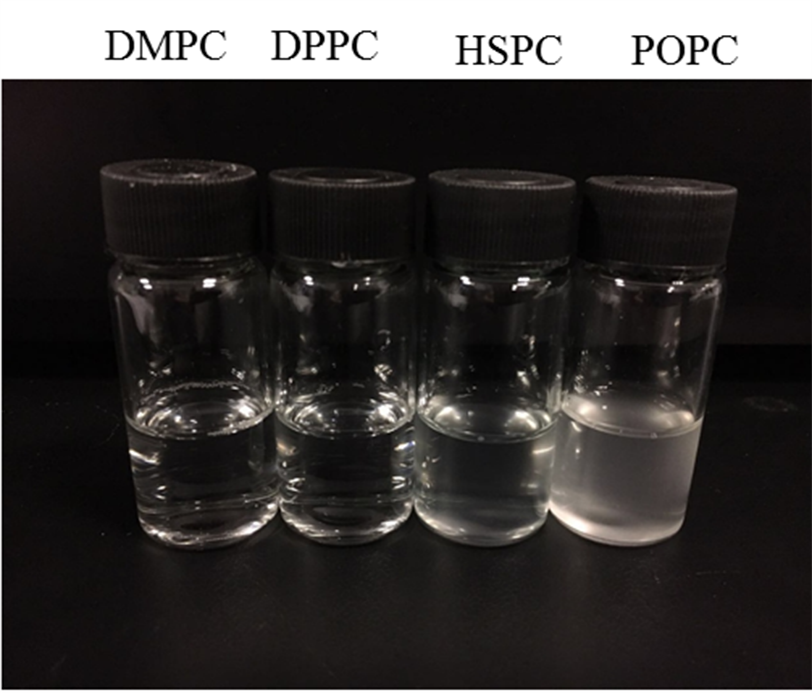


Figure S2. The appearance of different types of sHDL nanoparticles.


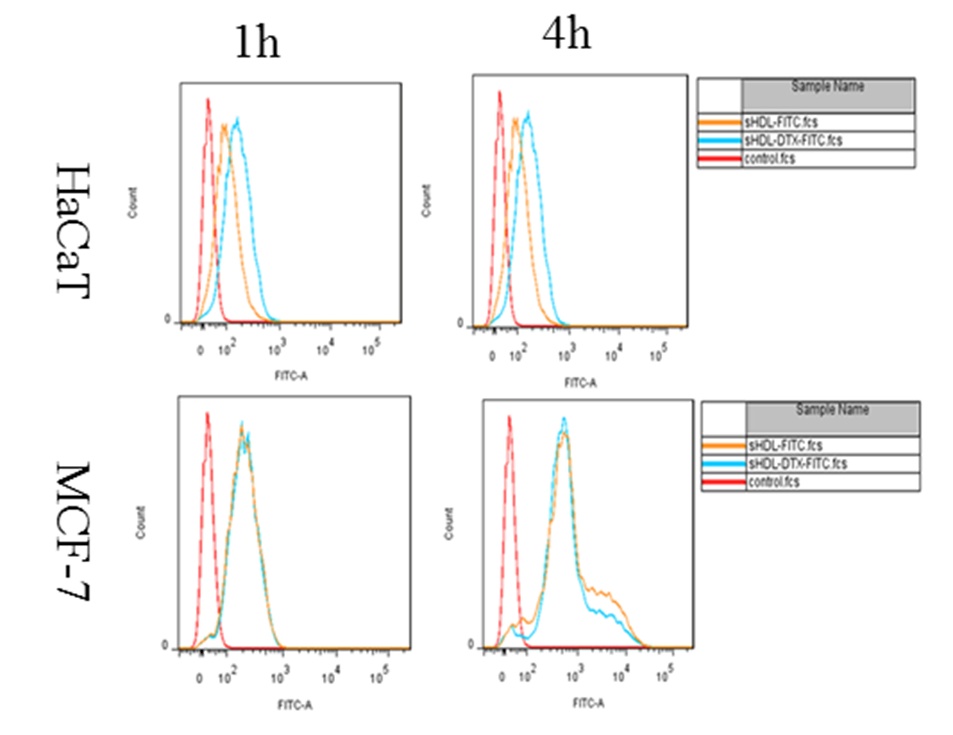


Figure S3. FCM results of cellular uptake in HaCaT cells and MCF-7 cells after incubation with sHDL-FITC nanoparticle and DTX-sHDL-FITC nanoparticle for 1 h and 4 h, respectively.


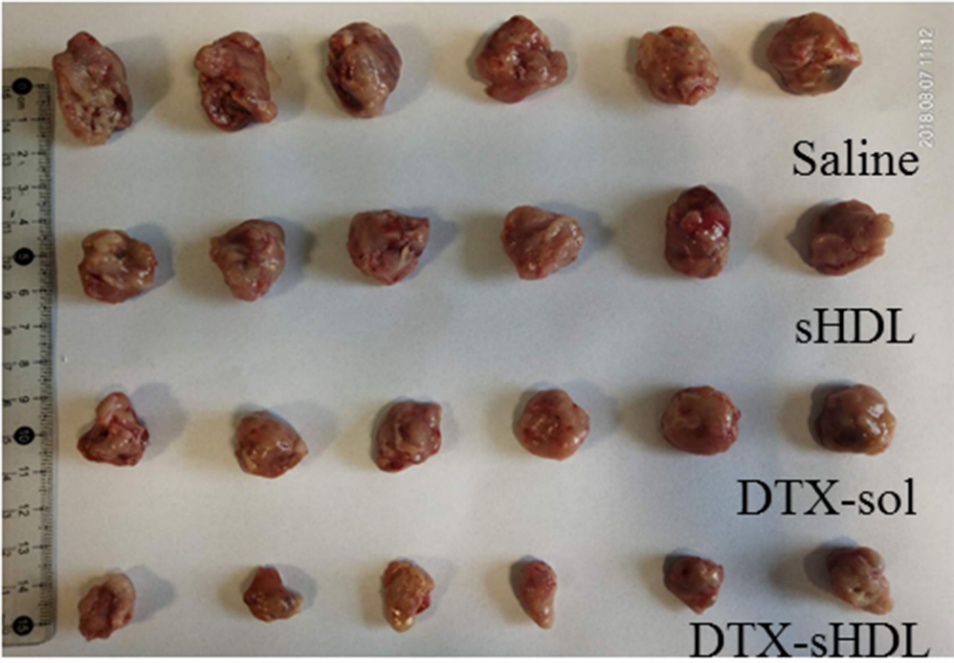


Figure S4. The image of resected tumor treated with Saline, DTX-sol, sHDL, and DTX-sHDL nanoparticles.

Table S1 The properties of blank sHDL made with different types of phospholipids

| Parameter | DMPC | DPPC | HSPC | POPC |
| --- | --- | --- | --- | --- |
| Size | 12.58±0.34 | 20.84±0.13 | 30.09±0.27 | 118.84±61.07 |
| Turbidity | 0.022±0.002 | 0.061±0.001 | 0.292±0.002 | 0.598±0.0006 |
| Enthalpy | -233.08 | -278.14 | -301.43 | -307.78 |
| Binding Energy | -357.798 | -320.962 | -259.296 | -281.279 |

Table S2 *In vitro* release kinetics parameters.

| Model | Equation | Equation pH 7.4 | R^2^ |
| --- | --- | --- | --- |
| Zero order | R=kt+C | R=0.0138t+0.2795 | 0.8785 |
| First order | In(1-R)=kt+C | In(1-R)= -0.057t-0.0639 | 0.9729 |
| Higuchi | R= kt**^1/2^** +C | R=0.1323t**^1/2^** +0.0342 | 0.9811 |
| Riger-Peppas | InR=kInt +C | InR=0.488Int -1.9129 | 0.9894 |
